# Porcine jejunal Peyer’s patches leukocytes are a potent source of mucosal IgA binding to *Streptococcus suis* and suilysin

**DOI:** 10.1186/s13567-026-01859-y

**Published:** 2026-10-29

**Authors:** Anne-Marie Seidel, Johanna Miriam Spitzer, Wieland Schrödl, Gabriele Köhler, Johannes Kauffold, Gottfried Alber, Christoph Georg. Baums, Maria Eschke

**Affiliations:** 1 Institute of Immunology, Centre for Infectious Diseases, Faculty of Veterinary Medicine, Centre for Biotechnology and Biomedicine, Leipzig University, Deutscher Platz 5, 04103 Leipzig, Germany; 2 Institute of Bacteriology and Mycology, Centre for Infectious Diseases, Faculty of Veterinary Medicine, Leipzig University, Leipzig, Germany; 3 Institute of Pathology, Klinikum Fulda gAG, Fulda, Germany; 4 Clinic for Ruminants and Swine, Faculty of Veterinary Medicine, Leipzig University, Leipzig, Germany

**Keywords:** *Streptococcus suis*, pig, IgA, Peyer’s patches, mucosal immunity, suilysin

## Abstract

Human *Streptococcus suis* infections are frequently linked to undercooked pork consumption, particularly in Southeast Asia. Experimental evidence indicates that *S. suis* can cross the intestinal barrier and cause systemic infection in pigs, suggesting the gut as a plausible entry site. However, mucosal immunity against *S. suis* in the intestinal tract remains poorly characterized. Here, we provide initial insights into the intestinal source and production of mucosal IgA against *S. suis* in 8-week-old conventional pigs. We show that jejunal and ileal mucus contains IgA recognizing a commensal *S. suis* isolate as well as a virulent *S. suis cps*2 strain. Mucosal IgA antibodies also bind to the secreted virulence-associated protein suilysin (SLY). Flow cytometric analysis of porcine Peyer’s patches (PP) leukocytes of the jejunum (JPP) and the ileum (IPP) revealed a significantly higher frequency of IgA^+^ B cells in JPP. Moreover, JPP-derived leukocytes showed higher constitutive and stimulation-induced total IgA secretion than IPP-derived leukocytes. Stimulation, either with the T cell-associated factors CD40L and IL-21 or the TLR1/2 ligand Pam3Cys-SK4 induced anti-*S. suis* IgA secretion, particularly in JPP-derived leukocyte cultures, indicating a contribution of innate immune mechanisms and an important role for T cell help. Finally, IgA induced by in vitro stimulation of JPP- or IPP-derived leukocytes recognized commensal and virulent *S. suis* as well as SLY, involved in disrupting epithelial barriers. Our results suggest that *S. suis* elicits a mucosal immune response in the intestinal tract of conventional piglets, with B cells in JPP representing a key site of IgA production.

**Supplementary Information:**

The online version contains supplementary material available at https://doi.org/10.1186/s13567-026-01859-y.

## Introduction

*Streptococcus suis* is a natural colonizer of the mucosal surfaces of healthy pigs. Its primary niche is the upper respiratory tract, particularly the tonsils and nasal cavities, although it is also detected in the urogenital and gastrointestinal tracts [1–4]. At the same time, *S. suis* is a zoonotic pathogen capable of causing severe, life-threatening disease—such as meningitis and septicemia—also in humans [5–7]. High morbidity in weaned piglets is one of the main reasons for the extensive use of antimicrobials at this age, because a licensed, cross-protective vaccine is not available. The polysaccharide capsule protects *S. suis* from phagocytosis and modulates the host immune response [11, 12]. To date, 29 distinct serotypes of *S. suis* have been identified, based on differences in their capsular polysaccharides (*cps*) [8, 9]. Although the distribution of *S. suis* serotypes varies geographically, *cps*2 is considered the most clinically relevant serotype in both pigs and humans worldwide [10]. In addition to the polysaccharide capsule, several surface-associated and extracellular proteins contribute to the pathogenicity of *S. suis* [11]. Among these, suilysin (SLY), a 54 kDa secreted hemolysin, belonging to the family of cholesterol-dependent cytolysins, can induce pores in various host cell membranes, resulting in cell lysis and tissue damage [12–15]. SLY is present in 95% of the European and Asian invasive *cps*2 strains [16, 17]. By inducing cytoskeletal rearrangements in epithelial cells, SLY enhances adhesive and invasive capacities of *S. suis* [18]. SLY also interferes with the host immune response by modulating cytokine production and impairing phagocyte function [19, 20], thereby promoting bacterial survival and systemic dissemination [21]. SLY is highly immunogenic, eliciting strong antibody responses, although the protective effect in pigs varies across studies [22, 23].

IgA is the predominant antibody isotype at mucosal surfaces and plays a crucial role in shaping the microbiome as well as in protecting against invasive mucosal infections [24–27]. IgA contributes to mucosal defense by blocking or sterically hindering microbial factors involved in epithelial attachment, mediating neutralization of invading pathogens and inflammatory microbial products (immune exclusion), and facilitating antigen sampling through interactions with microfold (M) cells, specialized epithelial cells responsible for antigen uptake [28, 29]. Peyer’s patches (PP) are primary inductive sites of gut-associated lymphoid tissue (GALT) and play a pivotal role in initiating mucosal immune responses, particularly in the induction of IgA-producing B cells [30]. Two anatomically distinct types of Peyer’s patches can be distinguished in pigs. The morphologically continuous ileal Peyer’s patch (IPP) is formed prenatally, whereas the discrete jejunal Peyer’s patches (JPP) develop after birth and expand significantly after weaning [31, 32]. These structural differences are associated with functional specialization [33], indicating distinct roles of IPP and JPP in shaping mucosal immunity. Consistent with this concept, gene expression profiling of porcine Peyer’s patches has provided molecular evidence supporting this functional division [33]. In neonatal piglets, colonization of the intestinal tract by commensal bacteria stimulates the expansion of IgA-positive B cells within Peyer’s patches [34, 35]. Accordingly, porcine Peyer’s patches may represent an important source of *S. suis*-binding IgA.

In South-East Asia, consumption of undercooked blood, meat and intestine of pigs was shown to be the main risk factor for zoonotic *S. suis* infections [36–38]. This finding led to a number of studies investigating whether the intestinal tract is also a putative entry site for *S. suis* infections in pigs [39, 40]. Although the intestinal route is not considered the primary route of colonization in pigs, studying the intestinal mucosal immune response in the natural reservoir host may provide mechanistic insight into the principal route of zoonotic transmission in humans. However, mucosal immunity in the intestinal tract against *S. suis* has, to the best of our knowledge, not been investigated. Based on these considerations, we hypothesized that Peyer’s patches are an important source of *S. suis*-specific IgA in weaning piglets. In this study, we show that JPP leukocytes of conventional piglets produce IgA binding to *S. suis* cps2 and SLY, suggesting that JPP may contribute to mucosal protection against intestinal exposure to SLY-secreting *S. suis* *cps*2.

## Materials and methods

### Bacteria and growth conditions

*S. suis cps*2 strain 10 is a virulent sequence type 1 (ST1) strain that secretes suilysin (SLY) and has repeatedly been shown to cause severe disease in experimental pig infection models [19, 41–43]. Streptococci were grown overnight at 37 °C in Bacto^™^ Todd-Hewitt-Broth (THB) (BD, Heidelberg, Germany) or on Columbia agar plates with 6% sheep blood (Thermo Oxoid, Schwerte, Germany). *S. suis* stocks were prepared at the exponential growth phase (optical density (OD)600 nm = 0.5) and stored at −80 °C in 15% glycerol as single-use aliquots. *Escherichia coli* M15 and BL21 carrying derivatives of pQE30 and pET45, respectively, were grown in LB medium with 100 µg/mL ampicillin.

*S. suis* Fx2VA18 was isolated from the chymus of the small intestine of one of the animals sampled for flow cytometric analysis of cells from Peyer`s patches in this study. The isolate was confirmed as *S. suis* by MALDI-TOF MS analysis using the Bruker Biotyper Library and detection of amplicons for *arc*A and *gdh* in a *S. suis* multiplex PCR (MP-PCR) [44]. Based on the results of the MP-PCR, strain Fx2VA18 does not carry the *sly, mrp* or *epf* genes and does not belong to *cps* 1, 2, 1/2, 7, 9 or 14. In addition, PCR according to Wileman et al*.* [45] confirmed Fx2VA18 as well as strain 10 as *S. suis* through the 722 bp SSU0577 amplicon. Strain Fx2VA18 was classified as non-disease-associated through the 892 bp SSUST30534 amplicon, while strain 10 tested positive for the disease-associated markers SSU0207 and SSU1589.

### Mucus collection and preparation

Intestinal mucus was collected from six 8-week-old conventionally bred and held German Landrace piglets. The pigs originated from the Teaching and Research Farm Oberholz, Großpösna, Germany.

Piglets were weaned at the age of 4 weeks, then moved into the nursery where they stayed in pens of up to 10 animals until 12 weeks of age. They were fed with a commercial pelleted diet ad libitum. Water was also supplied ad libitum through nipple drinkers. The herd carries multiple *S. suis cps* including *cps*1, *cps*1/2*, cps*2*, cps*7 and *cps*9 as determined by genotyping numerous *S. suis* isolates of tonsil swabs by multiplex (MP)-PCR [44] and sequencing of a *cps*K amplification product [46]. For this purpose, 15–30 cm segments of the aboral jejunum and ileum were excised, opened longitudinally, and gently rinsed with sterile phosphate-buffered saline (PBS) to remove ingesta. Tissue segments were then spread flat on a cutting board, and mucus was carefully scraped off using cell scrapers (Cell Scraper S, TPP, #99002) and collected in 1.5- or 2-mL tubes. Mucus samples were diluted 1:4 in PBS, vortexed for 20 s, and centrifuged at 12000 × *g* for 45 min at 4 °C. The supernatant was collected for further analysis.

### Recombinant *S. suis* proteins

Recombinant His-tagged SLY (rSLY) was expressed in *E. coli* BL21 isolates carrying plasmid pET45bslynew [23] and purified following induction with isopropyl-β-D-thiogalactopyranoside (IPTG) using Ni^2^⁺-nitrilotriacetic acid (Ni–NTA) affinity chromatography under native conditions, as previously described [42].

Recombinant surface antigen one (rSAO) [47], Immunoglobulin M-degrading enzyme of *S. suis* homolog (rIde_*Ssuis*__h) [48], and muramidase-released protein (rMRP) [43] were expressed and purified as previously described.

### Generation of rRhiA-I

Genomic DNA of *Rodentibacter heylii* SF27GVG was isolated using the DNeasy Blood & Tissue Kit (Qiagen, Hilden, Germany) according to the manufacturer’s instructions. One Taq (New England Biolabs, Ipswich, USA) and the primer pair rhiA_f-I (GATCAGCCCGGGACCTTGACGAAAGATGCAAATG) and rhiA_r-I (GATCAGCTCGAGGTTACCGTCAGCGTCTTTAG) were used to amplify nucleotides encoding aa 213–324 of the RhiA protein [49]. After digestion with *Xma*I and *Xho*I (restriction sites underlined above), this fragment of the *rhi*A gene was cloned into pET21b-JF containing sequences for an N-terminal Strep-tag and a C-terminal His-tag. The obtained plasmid pET21b-JF-RhiA-I was transformed into *E. coli* BL21 by electroporation, followed by recombinant expression of rRhiA-I through addition of 1 M IPTG for 4 h at 37 °C. Recombinant protein was purified by Protino Ni-TED kit (Macherey–Nagel, Düren, Germany) according to the manufacturer’s instructions. Finally, rRhiA_-_I was dialysed against PBS (pH 7.4) and protein concentration was measured with the Eon microplate spectrophotometer (BioTek, Bad Friedrichshall, Germany).

### SDS-Page and western blot analysis

SDS-Page and western blot analysis were performed to detect porcine IgA against *S. suis* proteins in intestinal mucus. Final amounts of 2 µg rRhiA-I, 2 µg rIde_*Ssuis*__h, 1 µg rSLY, and 0.25 µg rSAO were separated in 4% stacking gels and 8% separation gels. Protein staining was performed for 2.5 h in PageBlue Protein Staining Solution (Thermo Scientific, Rockford, USA, #24620) and destaining overnight under gentle shaking in demineralized water at 4 °C.

After semi-dry blotting of proteins onto a nitrocellulose membrane (Roth, Karlsruhe, Germany, #1YXN.1), the membranes were blocked overnight at 4 °C in TBS-T (Tris-buffered saline with 0.05% Tween 20) with 5% skim milk. All antibody incubations were performed in TBS-T with 1% skim milk at RT under constant shaking. Between all incubation steps membranes were washed at least 4 times in TBS-T. Mucus was used as a primary antibody source in a dilution of 1:100 (see above for mucus preparation). To detect bound IgA, 0.1 µg/mL of secondary HRP-conjugated rabbit anti-porcine IgA was added. For detection of secretory antibodies, a mouse anti-pig secretory component antibody (clone: K60 1F1; 0.5 µg/mL; Biorad, Feldkirchen, Germany) and a goat anti-mouse IgG tertiary antibody (0.08 µg/mL; Dianova, BIOZOL Diagnostica, Hamburg, Germany) were used. Proteins were analyzed using WesternBright™ Quantum chemiluminescence substrate (Biozym, Hessisch Oldendorf, Germany, #541014) according to manufacturer’s instructions. Detection was carried out using Fusion SL imaging system and FusionCapt Advance Solo 4 software (both by Vilber Lourmat, Eberhardzell, Germany). Marker bands were layered automatically.

### Enzyme-linked immunosorbent assay (ELISA)

Secretory IgA in porcine mucus samples was assessed by a sandwich enzyme-linked immunosorbent assay (ELISA). Nunc MaxiSorp^™^ flat bottom plates (ThermoFisher, Darmstadt, Germany) were coated overnight (O/N) at 4 °C with 1 µg/mL anti-pig IgA heavy chain antibody (A100-102A, Bethyl Laboratories). All incubation steps described below were separated by washes with phosphate-buffered saline containing 0.5% (v/v) Tween 20 (PBS-T). Non-specific binding sites were blocked with PBS supplemented with 5% bovine serum albumin and 0.1% gelatin for 1 h at room temperature. Prior to incubation, mucus samples (*n* = 6) were standardized to a defined total protein concentration. Wells were incubated with 3.5 ng of total protein per sample in PBS supplemented with 5% BSA, 0.1% gelatin, and 0.5% Tween-20 for 1.5 h at room temperature. Detection was performed using a mouse anti-pig IgA secretory component antibody (clone: K60 1F1; 0.5 µg/mL; Biorad, Feldkirchen, Germany), for 1.5 h at room temperature, followed by a secondary horseradish peroxidase (HRP)-conjugated polyclonal anti-mouse IgG1 antibody (0.08 µg/mL; Dianova, BIOZOL Diagnostica, Hamburg, Germany). For signal development, 3,3’,5,5’-Tetramethylbenzidine substrate (TMB; KPL, medac, Wedel, Germany) was added and incubated for 1 h (assay-optimized) in the dark. The enzymatic reaction was stopped with 50 µL phosphoric acid (H₃PO₄; Carl Roth, Arlesheim, Switzerland). The OD at 450 nm with a reference wavelength of 630 nm was measured on a Spectra-Max ABS ELISA reader with SoftMax Pro software v7.3.1 (Molecular devices, Munich, Germany). Blank-reduced ODs were reported and compared.

Total IgA was quantified in cell culture supernatants from PP (see below) and standardized mucus samples by sandwich ELISA as described above, with the following modifications: Purified swine IgA was used as a standard, and a horseradish peroxidase (HRP)-conjugated mouse anti-pig IgA antibody (clone: K61 1B4; Biorad, Feldkirchen, Germany) was used for detection. Total IgA concentrations in supernatants were calculated from the standard curve, whereas IgA concentrations in mucus samples were expressed relative to total protein. For signal development, 3,3’,5,5’-Tetramethylbenzidine substrate (TMB; KPL, medac, Wedel, Germany) was added and incubated for 30 min (assay-optimized) in the dark.

For detection of antigen-binding IgA in mucus and supernatant samples, Nunc MaxiSorp^™^ flat bottom plates (ThermoFisher, Darmstadt, Germany) were coated overnight (O/N) at 4 °C with either 1 × 10^7^ cfu/well of inactivated *S. suis cps* 2 strain 10 or *S. suis* isolate Fx2VA18, or with 50 µg/well of rSLY. Blocking and sample incubation were performed as described above. Detection was conducted using either 250 ng/mL of a HRP-conjugated mouse anti-pig IgA antibody (clone: K61 1B4; Biorad, Feldkirchen, Germany), or 125 ng/mL of a HRP-conjugated goat anti-pig IgA heavy chain antibody (A100-102P, Bethyl Laboratories). Blank-reduced ODs were obtained as described above.

### Purification of IgA and generation of porcine IgA-specific antibodies

All chromatographic purification procedures were carried out with the ÄCTA prime plus chromatography system (GE Healthcare, Little Chalfont, United Kingdom). Size exclusion chromatography (SEC) was performed with the HiLoad 16/600 Superdex 200 pg column (GE Healthcare via VWR, Darmstadt, Germany, catalogue 28−9893-35) under the following conditions: flow rate 1 mL/min, 2 mL sample load, collected eluate 2 mL/tube and phosphate buffered saline (PBS, pH 7.35) as running buffer. For affinity chromatography and pre-absorption steps, proteins were covalently bound to CNBr activated Sepharose 4B (GE Healthcare, Little Chalfont, United Kingdom, catalogue 17−0430-01) according to manufacturer instructions (instruction #71–7086-00 AF). Elution was performed with 0.1 M citric acid/sodium phosphate, pH 2.5 and the eluates were neutralized with 0.5 M trisodium phosphate. For purification of IgA from porcine gamma globulin fraction of sow´s milk, proteins from a 40% ammonium sulfate-saturated solution were precipitated with 6% polyethylene glycol 4000, and the resulting supernatant was subsequently precipitated with 14% polyethylene glycol 6000 (PEG6000, Carl Roth, Arlesheim, Switzerland, catalogue 0156). The 14% precipitate was then solubilized in PBS and purified by affinity chromatography with Sepharose columns covalently coupled to Protein G for IgG-removal (HiTrap protein G column, GE Healthcare via VWR, Darmstadt, Germany, catalogue 17040501), followed by affinity chromatography with HiTrap protein A column (GE Healthcare via VWR, Darmstadt, Germany, catalogue 17040301) and size exclusion chromatography with the concentrated (see below) Protein A-eluate. The eluates with corresponding size to IgA or sIgA were pooled and concentrated with 10 kDa cutoff centrifugal filters (VIVASPIN 20^®^, Sartorius, Göttingen, Germany, catalogue VS2001).

Purified milk IgA was used as an antigen for immunization of rabbits, as previously described [50]. Resulting anti-pig IgA was used in western blot analysis (see above).

### Preparation and isolation of leukocytes from porcine Peyer’s patches

Leukocytes were isolated from JPP and IPP of 8-week-old, healthy pigs, (*n* = 6) originating from the same herd used for intestinal mucus collection, as previously described (adapted from Solano-Aguilar et al. [70] with minor modifications. Briefly, jejunal and ileal segments were collected immediately post mortem and placed in ice-cold PBS. Peyer’s patches were excised from the intestinal tissue using sterile instruments while avoiding direct contact with the tissue surface. A total of 10 JPP and a 10 cm segment of the continuous IPP were collected per animal. Tissue samples were washed in PBS to remove luminal contents. Representative pieces of JPP and IPP were fixed in 4% paraformaldehyde for histological analysis. The remaining tissue was minced and transferred into 15 mL tubes containing cell medium (IMDM with L-Glutamine, 25 mM HEPES and 3.024 g/L NaHCO3; PAN-Biotech) supplemented with 10% heat-inactivated FBS, 100 U/mL penicillin, and 100 μg/mL streptomycin (both purchased from PAA Laboratories, Cölbe, Germany). For enzymatic digestion, 100 U/mL DNase (ThermoFisher, Darmstadt, Germany) and 15 U/mL collagenase (Roche, Grenzach-Wyhlen, Germany) were added freshly, followed by incubation for 30 min at 37 °C under rotation. Samples were then mechanically dissociated using the GentleMACS (Miltenyi, Bergisch Gladbach, Germany) and passaged through a 100 µm cell strainer (Th. Geyer, Renningen, Germany). Cells were washed with IMDM and centrifuged at 500 × *g* for 8 min at 4 °C. Erythrocytes were lysed using erythrocyte lysis buffer (150 mM NH₄Cl, 10 mM KHCO₃, 0.1 mM EDTA). Cells were washed and subsequently subjected to density gradient centrifugation. Briefly, cell suspensions were layered above Pancoll® Separating Solution (density 1.077 g/mL, PAN-Biotech, Aidenbach, Germany) and centrifuged (900 × *g*, 35 min at room temperature, minimal acceleration and deceleration). Mononuclear cells were collected from the interphase and washed in culture medium (IMDM with L-Glutamine, 25 mM HEPES and 3.024 g/L NaHCO3; PAN-Biotech) supplemented with 10% heat-inactivated FBS, 100 U/mL penicillin, and 100 μg/mL streptomycin (both purchased from PAA Laboratories, Cölbe, Germany) (500 *g*, 15 min, RT). Enriched leukocytes from Peyer’s patches were used immediately for phenotyping by flow cytometric analysis or cryo-preserved in liquid nitrogen until stimulation [freezing medium: 50% FBS, 40% IMDM, 10% dimethyl sulfoxide (DMSO, Merck, Darmstadt, Germany)].

### Phenotyping of purified cells from Peyer’s patches by flow cytometric analysis

Frequencies of CD3⁺, CD79a⁺, and CD79a^+^ IgA⁺ immune cells in JPP and IPP were determined by flow cytometry. A fixable viability dye (eFluor 506, Thermo Fisher Scientific, Carlsbad, USA) was used according to the manufacturer’s protocol to allow for discrimination between viable and dead cells. To prevent binding via Fc receptors, cells were incubated with heat-inactivated normal serum derived from pig (30% in PBS). All surface staining steps described below were performed for 20 min on ice in the dark and were separated by washing steps with FACS buffer (PBS, 3% FBS, 0.1% sodium azide (Merck, Darmstadt, Germany); 500 × *g*, 3 min, 4 °C). Detailed information on the primary antibodies used for flow cytometric staining is provided in Table 1. Cells were first incubated with anti-pig IgA, followed by incubation with a fluorescently labeled anti-mouse IgG1 secondary antibody. In the final surface staining step, cells were incubated with a fluorescently labelled antibody against the pan-T cell marker CD3 (Table 1). For intracellular staining of the pan-B cell marker CD79a (Table 1), cells were permeabilized using the FoxP3/Transcription Factor Staining Buffer Set (Thermo Fisher Scientific, Carlsbad, USA) according to the manufacturer’s protocol. After permeabilization, an additional blocking step with 30% heat-inactivated normal serum was done and cells were incubated with the specific anti-CD79a antibody for 30 min at RT. Following acquisition with a BD LSR Fortessa^™^ flow cytometer (Becton Dickinson (BD), Heidelberg, Germany) equipped with BD FACS Diva^™^ software version 9.7.1 (BD), data was analyzed using the FlowJo^™^10.10 software (BD). After exclusion of doublets and dead cells, cells from Peyer’s patches were gated on CD79a^+^ B lymphocytes and CD3^+^ T lymphocytes. Additionally, the frequency of CD79a^+^IgA^+^ B lymphocytes was analyzed. Adequate gating was performed according to fluorescence minus one (FMO) controls.

**Table 1:** **Antibodies used for flow cytometric analysis**

Specificity	Clone	Isotype	Labeling strategy	Fluorochrome	Source
IgA	K61 1B4	Mouse IgG1	Secondary antibody^a^	Brilliant Violet (BV)421	Bio-Rad
CD3ε	BB23-8E6-8C8	Mouse IgG2a	Directly conjugated	PE-Cy7	BD biosciences
CD21	REA940	Human IgG1	Directly conjugated	PE, APC	Miltenyi
CD79a*	HM47	Mouse IgG1	Directly conjugated	PE	BioLegend

^a^Rat anti-mouse IgG1 (clone RMG1-1), Brilliant Violet 421, BioLegend.

^*^Intracellular detection.

### Stimulation of leukocytes from Peyer’s patches

The leukocytes from Peyer’s patches of 8-week-old conventional pigs (see above) were seeded at a density of 5 × 10^5^ per well in culture medium containing 50 ng/mL IL-2 (BioLegend, San Diego, USA) into round-bottom 96-well cell culture plates (TPP, Trasadingen, Switzerland). To evaluate the IgA response of leukocytes from Peyer’s patches to Toll-like-receptor (TLR) stimulation and T cell-associated signals, cells were stimulated with 10 µg/mL Pam3Cys-SK4 (InvivoGen, Toulouse, France) or 1 µg/mL human CD40L plus 50 ng/mL human IL-21 (both: BioLegend), respectively. To promote cell sedimentation, plates were centrifuged at 400 × *g* for 2 min before start of culture [51]. Cells from Peyer’s patches were incubated for 7 days at 37 °C and 5% CO_2_.

### Histopathological analysis

For histopathological analysis, organs were fixed in 4% buffered formalin and embedded in paraffin. 4 µm sections were stained with hematoxylin and eosine. For immunohistological analysis the following antibodies were used: anti-human Bcl-6 (clone: GI191E/A8, Merck, Darmstadt, Germany), CD3 (clone: 2GV6, Roche, Grenzach-Wyhlen, Germany) and Ki-67 (clone: 30-9, Roche, Grenzach-Wyhlen, Germany), all cross-reactive with the respective porcine antigens.

### Collection of serum and milk

Serum samples were drawn from the same six 8-week-old piglets from which Peyer’s patches were isolated for leukocyte preparation.

Milk was collected from sows of a different herd within a previous study [52], 10 and 20 days after farrowing. For IgA purification, milk samples from different animals and time points were pooled.

### Statistical analysis

Statistical analysis was performed using GraphPad Prism 10.2.0 (GraphPad Software Inc., San Diego, USA). The Shapiro–Wilk test was applied to test for normal distribution. Normally distributed data is presented with means. Nonparametric data is shown with medians. Depending on normal distribution, the paired t-test or the Wilcoxon matched-pairs signed-rank test (both: two-tailed) were used for statistical analysis. To correct for baseline IgA secretion, medium values of supernatants were subtracted from stimulated conditions for each sample prior to analysis.

## Results

### Mucosal IgA in the small intestine of conventional weaned pigs binds to commensal and virulent ***S. suis*** and SLY

**Figure 1:**
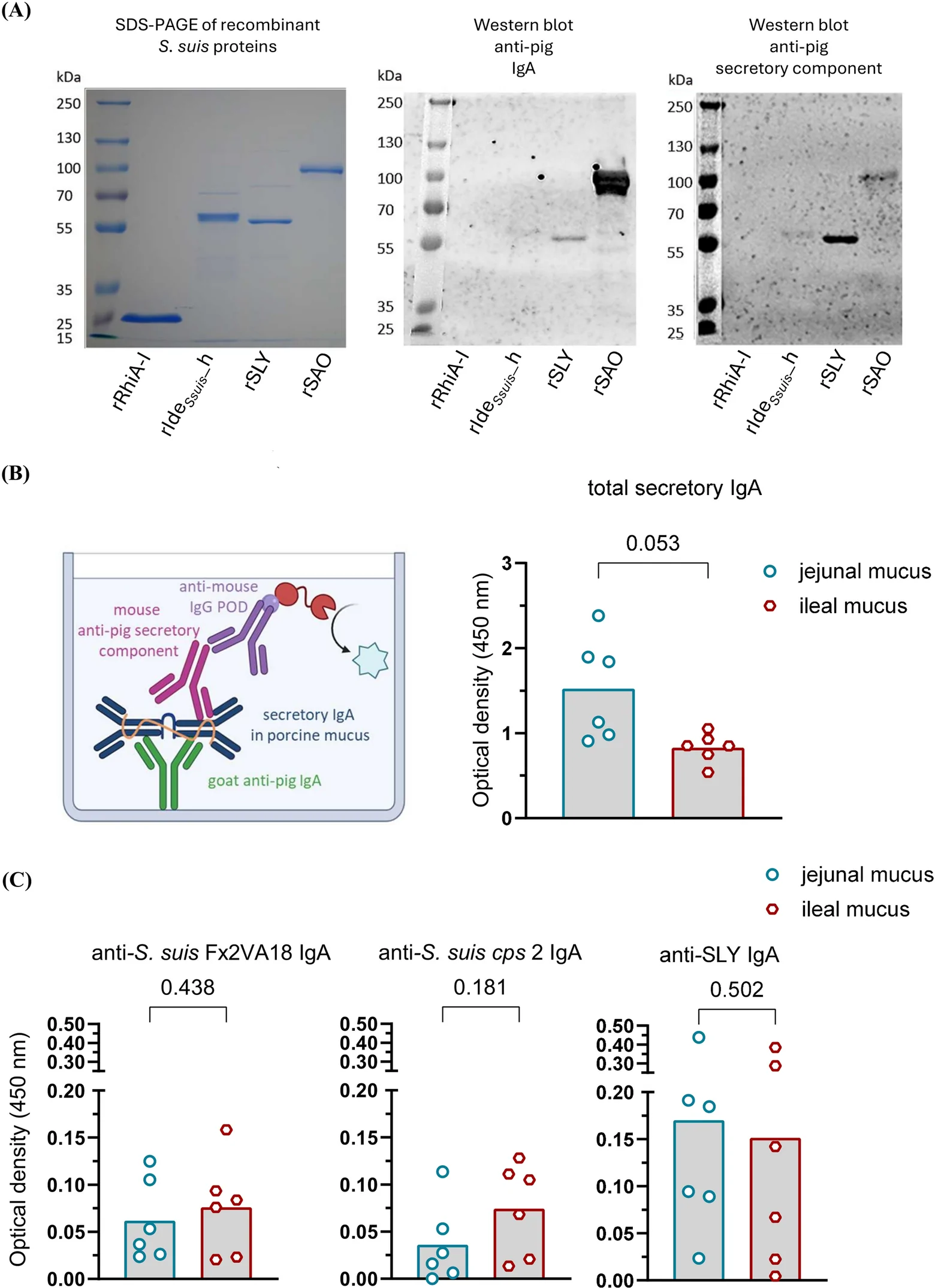
**Porcine intestinal mucus contains IgA binding to different** ***S. suis*** **antigens including suilysin.** A Western blot showing mucus IgA binding to different recombinant (r) *S. suis* proteins (rIde_*Ssuis*__h: recombinant Immunoglobulin M-degrading enzyme of *S. suis* homolog, SAO: surface antigen one, SLY: suilysin). As a bacterial antigen from a rodent pathogen but irrelevant to pigs, rRhiA-I (*Rodentibacter heylii* immunogen A fragment I) served as a negative control. **B** Experimental set-up to determine total secretory (S)IgA in porcine ileal and jejunal mucus by sandwich ELISA. Plates were coated with anti-IgA, and equal amounts of total protein per mucus sample, normalized prior to incubation, were applied per well. Detection was performed with a monoclonal anti-secretory component antibody and polyclonal POD-conjugated secondary antibodies. Total SIgA in ileal and jejunal mucus samples of 8-week-old pigs (*n* = 6) was detected as schematically depicted. Blank-reduced optical density (OD) values for mucus samples normalized to equal total protein are shown. Each dot represents one individual pig, the horizontal bars indicate mean values. Paired t-test was performed for statistical analysis. **C** Jejunal and ileal mucus samples of 8-week-old pigs were analyzed for IgA binding to the intestinal commensal *S. suis* isolate Fx2VA18, the virulent *S. suis cps*2 strain 10, and SLY by ELISA, using whole inactivated bacteria or recombinant SLY as coating antigen. Blank-subtracted OD values for mucus samples normalized to equal total protein are shown. Each dot represents one individual pig, the horizontal bars indicate mean values. Paired t-test (two-tailed) was performed for statistical analysis.

As previous studies proposed that *S. suis* crosses the intestinal barrier to cause invasive infection [39, 40, 53] we initially investigated if small intestinal mucus of conventional 8-week-old pigs contains specific IgA and secretory antibodies binding to different recombinant proteins of *S. suis* by western blot analysis (Figure 1A). A recombinant protein from a rodent bacterial pathogen, *Rodentibacter heylii* immunogen A (rRhiA), irrelevant for pigs, was used as a negative control. Recombinant bacterial proteins were immobilized on nitrocellulose membranes and incubated with mucus samples, followed by detection of bound IgA or secretory component. Small intestinal mucus IgA or secretory (S) antibodies (SIgA and SIgM) showed no reactivity toward rRhiA, indicating IgA binding selectively to porcine pathogens such as *S. suis* or *S. suis*-derived factors. In contrast, strong IgA and secretory antibody binding were observed to the *S. suis* proteins surface antigen one (SAO) and suilysin (SLY) (Figure 1A). The Immunoglobulin M-degrading enzyme of *S. suis* (Ide_Ssuis_) showed also low recognition by IgA and secretory antibodies (Figure 1A). Thus, multiple *S. suis* proteins were recognized by small intestinal mucus IgA and secretory antibodies in the investigated 8-week-old piglets.

Next, SIgA in mucus preparations of the jejunum and the ileum of six 8-week-old pigs was determined by sandwich-ELISA (Figure 1B). Intestinal mucus samples, normalized for total protein, were applied to anti-IgA-coated plates and the secretory component was detected (Figure 1B). SIgA was detectable in all samples with a trend toward higher optical densities in jejunal mucus (Figure 1B). In contrast to intestinal mucus (Figure 1B) and milk (Additional file 1), serum contained only low levels of total SIgA (Additional file 1), as expected [54]. This comparison demonstrates the enrichment of SIgA in intestinal mucus and supports the use of mucus samples to characterize the mucosal anti-*S. suis* IgA response.

Jejunal and ileal mucus samples of 8-week-old pigs were analyzed for IgA binding to both, commensal and virulent *S. suis* strains using whole inactivated bacteria as coating antigen (Figure 1C). IgA binding to the intestinal commensal *S. suis* strain was detected in ileal and jejunal mucus (Figure 1C). Similarly, IgA of all samples bound to virulent *S. suis cps*2 strain, with no significant differences observed between the intestinal regions (Figure 1C). Moreover, small intestinal mucus IgA-reactivity to the virulence factor SLY was confirmed by ELISA (Figure 1C). Overall, small intestinal mucus of the investigated piglets contains IgA binding to antigens from commensal and virulent *S. suis* strains, including SLY. This finding made us investigate Peyer`s patches (PP) as a putative source of IgA directed against *S. suis* antigens.

### IgA^+^ B cells and CD3^+^ T cells are enriched in JPP relative to IPP

**Figure 2:**
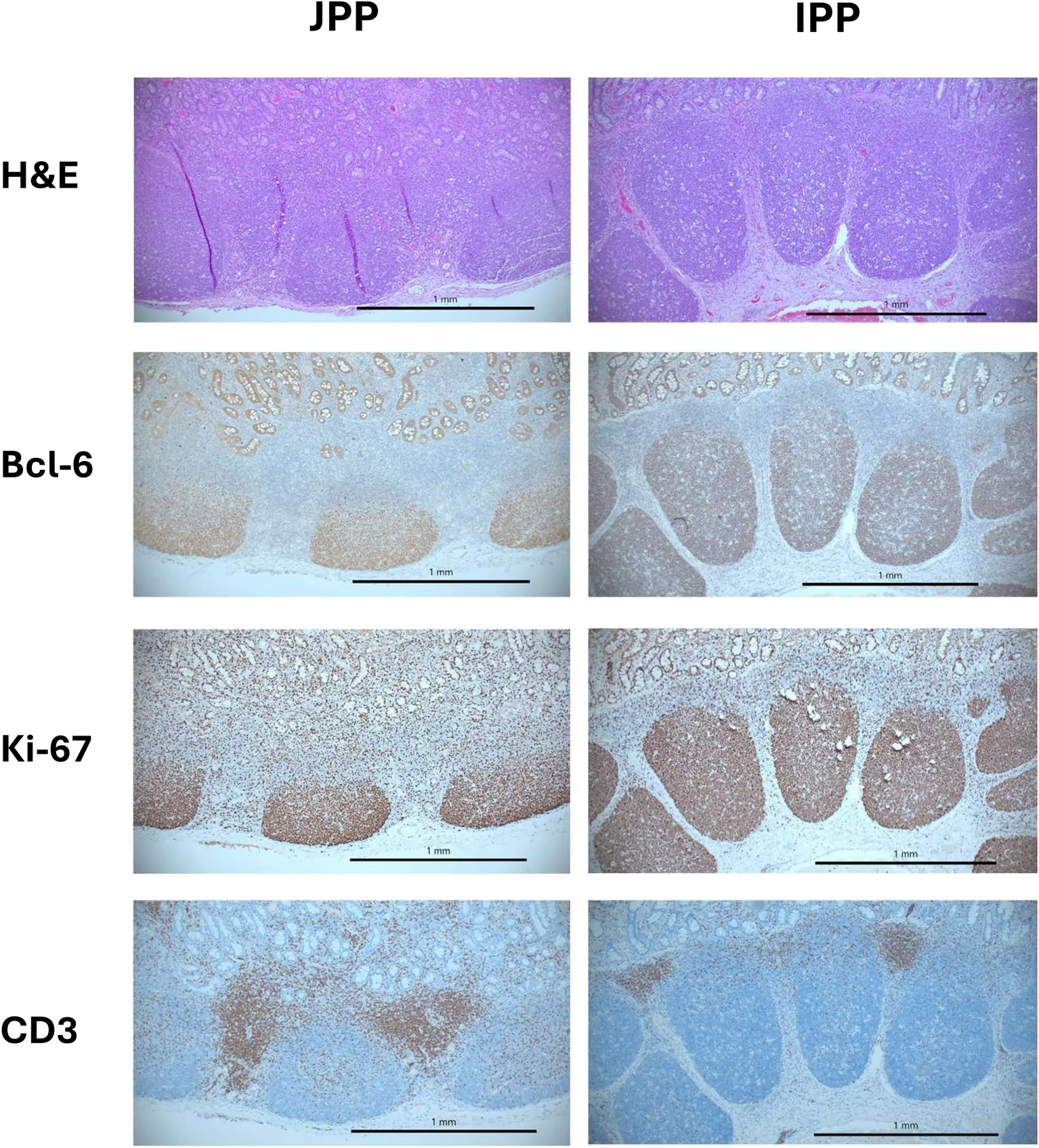
**Jejunal Peyer’s patches show higher T cell density but similar follicular structure with ileal patches.** Representative sections of JPP and IPP of an 8-week-old pig (pig with mean frequencies selected from Figure 3B) are shown. Hematoxylin and eosin (H&E) staining reveals intestinal mucosa with prominent PP. Immunohistochemical staining for Bcl-6, Ki-67, and CD3 identifies germinal center B cells, proliferating cells, and T cells, respectively.

**Figure 3:**
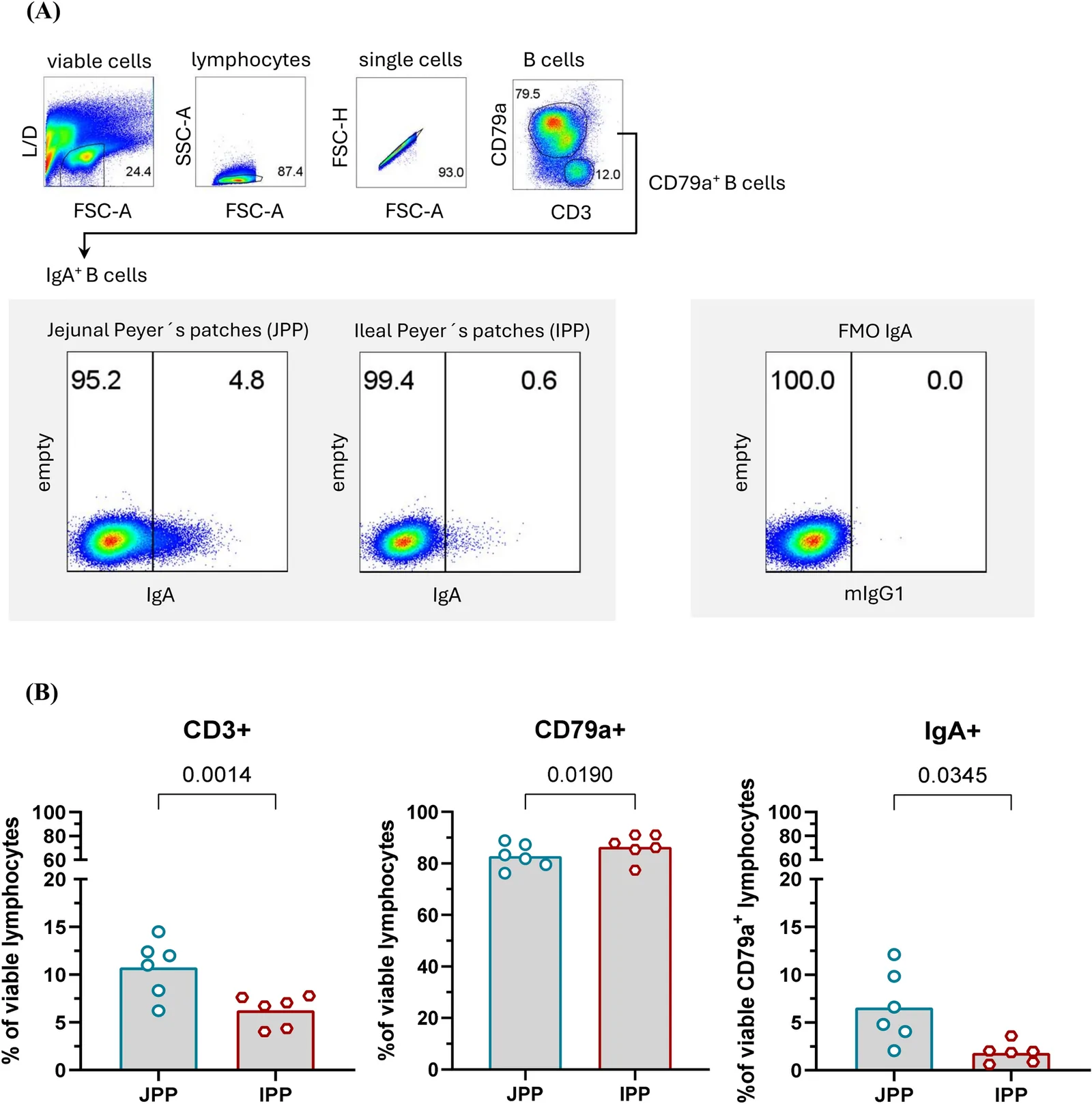
**Porcine jejunal Peyer’s patches contain a higher frequency of IgA**^**+**^** B cells than ileal Peyer’s patches. A** The frequency of IgA^+^ B cells in jejunal and ileal Peyer’s patches was determined in 8-week-old pigs by flow cytometry. Representative pseudocolor plots show the gating strategy: After exclusion of doublets and dead cells, CD79a^+^ B lymphocytes were selected to determine the frequency of IgA^+^ B cells in IPP and JPP. FMO fluorescence minus one control. **B** Quantification of CD79a^+^, CD3^+^ and CD79a^+^IgA^+^ lymphocytes among viable single cells in jejunal and ileal Peyer’s patches. Each dot represents one individual pig, the horizontal bars indicate mean values. Paired t-test was performed for statistical analysis.

Prior to functional characterization, we compared the histological organization of JPP and IPP of 8-week-old pigs. IPP and JPP showed a comparable follicular organization (Figure 2). Within the follicles of both tissues, Bcl-6⁺ cells were densely localized, consistent with well-organized germinal centers, and Ki-67 staining indicated high proliferative activity (Figure 2). CD3⁺ T cells appeared to be more abundant in JPP, localizing both within the follicles and in the apical interfollicular regions, suggesting a higher overall T cell density compared to IPP (Figure 2). To quantify T and B cell populations in IPP and JPP, flow cytometric analysis was performed. Representative results illustrating the gating strategy are depicted in Figure 3A. After excluding dead cells and doublets, T and B cells were identified based on CD3 and CD79a expression, respectively, and IgA expression was assessed within the CD79a⁺ B cell population. Consistent with the histological data (Figure 2), a significantly higher frequency of CD3^+^ T cells was detected in JPP compared with IPP (Figure 3B). As suggested by histology (Figure 2), CD79a⁺ B cells constituted a high proportion of viable lymphocytes in both tissues, though their relative frequency was slightly higher in IPP than in JPP (Figure 3B). Interestingly, within the B-cell compartment, the proportion of IgA⁺ cells was significantly higher in JPP compared with IPP, indicating an enrichment of IgA⁺ B cells in jejunal Peyer’s patches (Figure 3B).

### Enhanced constitutive and stimulated IgA secretion by leukocytes from JPP compared with IPP

**Figure 4:**
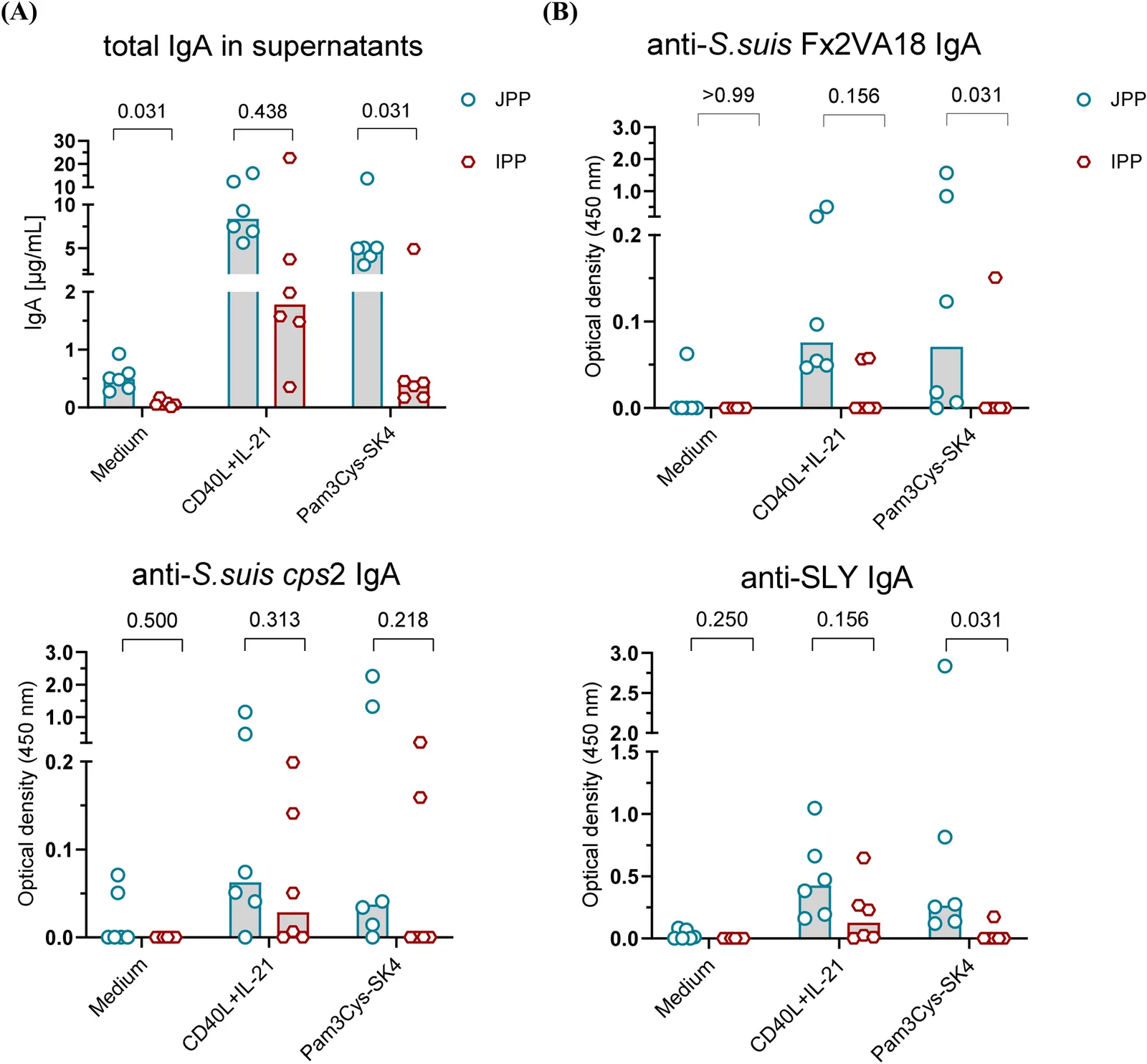
**Jejunal Peyer’s patches leukocytes are a potent source of IgA binding** ***S. suis*** **and suilysin.**Leukocytes from ileal Peyer’s patches (IPP) and JPP of 8-week-old pigs were incubated for 7 days in medium or stimulated with either the TLR ligand Pam3Cys-SK4 or the T cell-associated factors CD40L+IL-21. Supernatants were analyzed for total IgA by ELISA (**A**). Note enhanced constitutive and stimulated total IgA secretion by leukocytes from JPP compared with IPP. **B** Supernatants were analyzed for IgA binding to the intestinal commensal *S. suis* isolate Fx2VA18, the virulent *S. suis cps*2 strain 10, and suilysin (SLY) by ELISA, using whole inactivated bacteria or recombinant SLY as coating antigen. Blank-subtracted OD values are shown. Each dot represents one individual pig, the horizontal bars indicate median values. Wilcoxon matched-pairs signed rank test was performed for statistical analysis. To correct for baseline IgA secretion, medium values of supernatants were subtracted from stimulated conditions for each sample prior to analysis.

To assess IgA secretion, leukocytes from JPP and IPP were incubated in medium for 7 days or stimulated with either the Toll-like receptor (TLR) 1/2 ligand Pam3Cys-SK4 or with the T cell-associated factors CD40L and IL-21 [55]. Total IgA in supernatants was determined by ELISA. To consider baseline IgA secretion, values measured under medium conditions were subtracted from the corresponding stimulated conditions for each individual sample and coating. Noteworthy, leukocytes from JPP constitutively secrete IgA, whereas IPP leukocytes produced only negligible levels (Figure 4A). Stimulation with CD40L+IL-21 increased IgA secretion in both groups (Figure 4A). Similarly, stimulation with Pam3Cys-SK4 induced IgA secretion with a trend toward higher levels observed in supernatants of JPP cultures compared with IPP cultures (Figure 4A). Taken together, JPP-derived leukocytes constitute a potent source of IgA. As expected for supernatants from isolated IPP and JPP leukocyte cultures, the detected total IgA did not contain SIgA in contrast to IgA found in the intestinal mucus (Additional file 1).

### Leukocytes from JPP are a potent source of IgA against *S. suis* and SLY upon stimulation

Culture supernatants of in vitro-stimulated JPP and IPP-derived leukocytes were analyzed for *S. suis*-binding IgA. Under medium culture conditions, IgA binding to the commensal *S. suis* strain was largely undetectable in supernatants, but stimulation resulted in measurable optical densities, indicating induction of antigen-specific IgA (Figure 4B). Importantly, stimulation resulted in overall higher optical densities in supernatants from JPP-derived leukocytes compared with IPP-derived leukocytes, suggesting increased secretion of commensal *S. suis*-binding IgA (Figure 4B). This difference reached statistical significance for stimulation with Pam3Cys-SK4. A similar pattern was observed for CD40L+IL-21; however, variability between donors likely contributed to the lack of statistical significance. Stimulation also induced IgA binding to virulent *S. suis cps*2 and the virulence factor SLY, with generally higher optical densities in JPP-derived supernatants (Figure 4B). However, differences were only statistically significant for Pam3Cys-SK4-induced IgA binding to SLY, likely due to high inter-individual variability found for intestinal total IgA concentration (Additional file 2). Nevertheless, the data suggest that leukocytes from JPP represent a potent source of *S. suis*-specific IgA, capable of robust secretion upon stimulation.

## Discussion

The present study demonstrates that a mucosal IgA against *S. suis* is already present in the intestine of 8-week-old conventional pigs. IgA detected in intestinal mucus showed clear reactivity against multiple *S. suis* antigens, including the virulence-associated toxin SLY, as well as against inactivated commensal and virulent strains. These findings suggest that the porcine intestinal immune system mounts an IgA antibody response to *S. suis* early in life, at a time when piglets are highly susceptible to invasive disease.

Early mucosal anti-*S. suis* IgA appears to be self-generated rather than maternally derived. Passively transferred milk SIgA has been shown to have only a short half-life in the intestinal lumen of mice [56]. Moreover, a recent study in pigs reported a decline in maternally derived serum IgG over time, concomitant with an increase in serum IgA binding to the surface of *S. suis* [57]. The presence of antigen-binding IgA in intestinal mucus together with the observed potent production especially by JPP leukocytes supports the notion of a locally induced mucosal immune response against *S. suis*.

Epidemiological and experimental studies indicate that *S. suis cps2* of clonal complex 1 is a food-borne pathogen in humans [58, 59] and capable of translocating through the intestinal epithelium of pigs [39, 40, 53, 60, 61]. In this context, the cytolytic toxin SLY, produced by most invasive *S. suis* strains, including *cps*2 isolates, may contribute to translocation by disrupting epithelial barrier integrity, inducing cytoskeletal rearrangements and promoting bacterial adhesion and invasion [3, 11, 20]. Highly virulent *S. suis* strains, including those associated with streptococcal toxic shock syndrome (STSS), express elevated levels of SLY [21, 62, 63]. The strong IgA reactivity against SLY observed in this study suggests that this virulence factor is a major target of the early mucosal immune response in pigs. SLY-binding IgA in intestinal mucus may serve as an important first line of defense. By binding to this virulence-associated factor, IgA may limit its biological activity and thereby reduce epithelial damage as well as bacterial adherence and invasion. Thus, mucosal IgA may contribute to antimicrobial defense not only by targeting bacterial surface structures but also by neutralizing secreted toxins critical for pathogenesis. Our study provides the basis for further functional analysis to determine the extent to which SLY-binding IgA contributes to protection against invasive *S. suis* infection.

The site-specific characterization of immune cell composition in JPP and IPP in the present study extends previous observations of higher T cell frequencies in JPP and slightly increased B cell proportions in IPP [64, 65], by further defining the B cell compartment. We demonstrate a significantly higher frequency of IgA⁺ B cells in JPP, indicating functional differences between the IPP and JPP**.** This finding is consistent with the distinct developmental and functional specialization of both Peyer’s patch compartments [32]. While CD79a is expressed throughout B-cell development and therefore mainly reflects the overall B-cell compartment, IgA expression identifies antigen-experienced, class-switched B cells. Previous studies have shown that jejunal Peyer’s patches preferentially support antigen-driven B-cell differentiation and IgA class switching [33, 66, 67], whereas ileal Peyer’s patches are thought to primarily contribute to the establishment of the early, largely antigen-independent B-cell repertoire [35]. Functionally, JPP-derived cells exhibited significantly elevated constitutive IgA secretion, consistent with a more activated or primed baseline state compared with IPP, and showed an enhanced responsiveness to CD40L + IL-21 stimulation, likely reflecting T cell-dependent mechanisms. In our experimental setup, PP-derived leukocytes were cultured as mixed populations containing both B and T cells, thereby preserving physiologically relevant cellular interactions. This is particularly relevant given that isolated B cells, as well as PP-derived cells in general, have been reported to exhibit poor survival in vitro [65, 68]. Accordingly, while JPP-derived cells showed strong responsiveness to TLR stimulation, indicating a substantial contribution of innate signals to B cell activation, their enhanced overall responsiveness likely reflects a synergistic interplay between T cell-dependent and T cell-independent mechanisms within the mixed leukocyte population, as previously suggested [69].

T cell dependency of mucosal *S. suis*-binding IgA⁺ B cells supports their contribution to adaptive immune responses in weaned piglets. Notably, an increase in serum anti-*S. suis* IgA as well as bactericidal immunity post weaning suggests a potential role of IgA in systemic immunity [57].

In summary, this study identifies the intestine as an immunologically active site of early *S. suis* recognition and highlights JPP as a potent inductive site for anti-*S. suis* IgA responses. Our observations suggest that T cell help and innate signals may shape the generation of IgA capable of targeting *S. suis* and associated virulence factors such as SLY. From a translational perspective, the results of this study may have important implications for vaccine development and disease prevention. Given the apparent role of the intestine as a potential entry site and the early presence of antigen-specific IgA, strategies aimed at enhancing mucosal immunity may be particularly effective. Specifically, targeted stimulation of IgA⁺ B cells in Peyer’s patches, both in pigs and potentially in humans, could help to prevent intestinal infection with *S. suis*. Mucosal vaccination approaches that induce robust IgA responses against key virulence factors such as SLY may therefore represent a promising avenue to reduce both animal disease burden and zoonotic transmission.

## Supplementary Information

**Additional file 1: Total secretory IgA in intestinal mucus and cell culture supernatants. **Total secretory IgAin porcine ileal and jejunal mucus samples, supernatants from stimulated JPP-derived leukocytes, serum, sow milk, and purified milk IgA was determined by sandwich ELISA.

**Additional file 2: Total IgA concentrations in porcine jejunal and ileal mucus. **Total IgA concentrations in jejunal and ileal mucus samples of 8-week-old pigs were determined by sandwich ELISA.

## Data Availability

The datasets used and/or analyzed during the current study are available from the corresponding author upon reasonable request.
